# Supplementary material for: Using shape to turn off blinking for two-colour multiexciton emission in CdSe/CdS tetrapods
Source: Nat Commun. 2017 May 12;8:15083. doi: 10.1038/ncomms15083 (PMC5437295; doi:10.1038/ncomms15083)
Supplement: Supplementary Information — Supplementary Figures, Supplementary Tables, Supplementary Notes and Supplementary References [file ncomms15083-s1.pdf]

Supplementary Note 1: Synthetic methods and basic characterization of tetrapod shape series

Materials. Cadmium oxide (CdO, 99.5%), 1-octadecene (ODE, 90%), sulfur (reagent grade), selenium (99.99%, 100 mesh powder), oleic acid (OA, 90%), oleylamine (technical grade, 70%), and myristic acid (90%) were purchased from Sigma Aldrich. Trioctylphosphine (TOP, 97%) was purchased from Alfa Aesar, n-octadecylphosphonic acid (ODPA, 97%), trioctylphosphine oxide (TOPO, 99%) and n-hexylphosphonic acid (HPA, 97%) were purchased from Strem. All chemicals were used without further purification.

Synthesis of zinc blende (zb) CdSe quantum dot (QD) "seeds" for tetrapod growth. zb-CdSe QDs were synthesized via a previously reported method.[1] In a 50 mL three-neck round bottom flask 0.3 mmol CdO, 0.6 mmol myristic acid and 5 mL of 1-ODE were degassed at 90 °C for ~1 h. The resulting mixture was then heated to 250 °C for ~10-15 min to yield a clear solution, followed by the addition of 12 mL of ODE before cooling to 90 °C to degas for another hour. Upon cooling to room temperature, 0.012 g (0.15 mmol) Se was added to the reaction mixture and degassed at 50 °C for ~20 min. Upon heating to 240 °C under argon, color changes from colorless to yellow at ~150 °C and then to orange-red at 240 °C were observed, signifying the formation of zb-CdSe QDs. A degassed mixture of 0.5 mL oleic acid and 0.5 mL oleylamine in 2 mL of 1-ODE was subsequently added dropwise to the reaction mixture. The growth time for a ~4 nm diameter QD was approximately 2 h. The QDs were precipitated from the growth solution by adding acetone, and then subjected to two cycles of suspension and precipitation using toluene and methanol, respectively. The processed QDs were dispersed in a minimum amount of toluene and their concentration determined by measuring their absorbance at 350 nm, whose molar absorptivity is

known.[2] The toluene was then removed under vacuum and TOP was added to yield a QD concentration of 100 μM (stock QD solution).

Seeded CdSe/CdS tetrapod synthesis. CdSe/CdS tetrapods were synthesized following previously reported methods using the seeded growth technique.[3,4] Briefly, 2.65 g TOPO, 0.052 g CdO and a mixture of ligands selected for fine-tuning of the arm diameter and length (see **Supplementary Table 1**) were degassed at 150 °C for ~1.5 h in a 50 mL three-neck round bottom flask. The reaction mixture was then heated to 350 °C under Ar, whereupon the solution turned from reddish brown to colorless. Separately, a mixture of S-TOP and CdSe QDs was prepared by first dissolving S in TOP at 50 °C (7 mg in 0.6 mL) and then adding CdSe QD stock solution (~10^{-9} mol QDs). Upon reaching the injection temperature of 350 °C, an additional 1.8 mL of TOP was added, and the temperature was allowed to recover to 350 °C before the mixture of S-TOP and CdSe QDs was swiftly injected. The temperature was again allowed to recover to 350 °C and CdS arm growth continued for 10 min. The heating mantle was then removed and the solution was allowed to cool to 80 °C. The product was processed using repeated cycles of precipitation in methanol and re-dispersion in toluene. A crude approximation of the concentration of the processed CdSe/CdS tetrapods was determined using a previously reported procedure.[5]

Supplementary Table 1. Synthesis conditions used for tetrapod geometry tuning.

CdSe/CdS tetrapods	Ligands added
TP1: Thin/short<br>(Main text Figure 1a)	100 mg ODPA<br>0.5 mL OA
TP3: Thin/long<br>(Main text Figure 1c)	150 mg ODPA<br>0.5 mL OA
TP4: Thick/long<br>(Main text Figure 1d)	100 mg HPA<br>0.5 mL OA

Successive ionic layer adsorption and reaction (SILAR) shell growth on CdSe/CdS tetrapods. A variation of the SILAR shell-growth technique of Peng and co-workers[6] was used to overcoat thin/short (TP1; see main text) CdSe/CdS tetrapods to obtain thick/short (TP2) tetrapods.[7] Briefly, in a 50 mL round bottom flask was charged with an amount of washed CdSe/CdS T1 tetrapods (~10^{-7} mol), 2.5 mL of oleylamine and 2.5 mL of 1-ODE, and degassed at 60 ºC for 1 h. The mixture was heated to 240 °C under argon flow. Separately, stock solutions of 0.2 M S in ODE and 0.2 M Cd-oleate in ODE (1:4 molar ratio Cd:oleic acid) were prepared and used as precursors for shell growth. Five monolayers of CdS were grown at 240° C onto CdSe/CdS tetrapods with alternating additions of Cd and S precursors (calculated in each case to add one monolayer at a time; 2 h and 1 h anneal times were used between additions of Cd and S, respectively).

Basic optical and structural characterizations. Absorbance was measured using a CARY UV-Vis-NIR spectrophotometer, while photoluminescence and photoluminescence excitation spectra (excitation swept from 400-660 nm for emission at 680 nm) were recorded using a Horiba NanoLog spectrofluorometer. Transmission electron microscopy (TEM) images were obtained using an FEI Tecnai F30 transmission electron microscope.

Calculating tetrapod volumes. The volume of a truncated cone was used to "estimate" volume for TP1, TP3 and TP4, while the volume of a cylinder was used to estimate volume for TP2. Inputs for these volume calculations (arm diameters and arm widths) came from our measurements of TEM images, with one exception. Specifically, to calculate the volume of a truncated cone (i.e., a cone that ends with a flat tip rather than a point, which we believe more accurately represents the shape of our TP1, TP3 and TP4 tetrapods), both the large-end "base" radius and the small-end

“tip” radius are required, along with cone height (arm length). We determined the values for base radius and cone height directly from TEM measurements. These quantities are provided, along with standard deviations generated by measuring 90-120 arms for each of the TP geometries in the caption for Figure 1 and Supplementary Table 2.

In contrast to arm base radius, we did not feel confident in our measurements of arm tip radius using TEM images, as the ends of the tetrapods often deviated in shape from that anticipated by an even progression of the cone from base to end. In particular, the very ends of the arms were often suddenly narrower than the segments of arm nearest to the tips. For this reason, we chose, instead, to measure the mid-point of the arms (again, using 90-120 arms; Supplementary Table 2). Assuming a roughly even progression from base to tip for the arm shape, the calculated tip radius was determined by following the lines created by connecting base-point and mid-point diameter lines – each drawn perpendicular to and centered on a line representing arm length at this line’s end and middle, respectively – to the full length of the arm. Given that the actual tips are smaller than that estimated in this way (Supplementary Table 2), the calculated volumes are likely an overestimate of the actual volumes, but as the arms taper at a relatively even grade for the majority of the arm length, we feel that our approach provides a reasonable estimate of arm volume. However, given the simplifications that we have necessarily made with respect to arm shape, we do not feel that the volumes that we report warrant inclusion of statistics, i.e., inclusion of standard deviations, as doing so would imply greater confidence in these values than is appropriate due to the clear variability in shape from arm to arm and the deviation from perfect truncated cone geometry.

Supplementary Table 2. Measured and calculated tetrapod arm size parameters.

Tetrapod	Measured arm diameter at base	Measured arm diameter at mid-point	Measured arm length	Calculated arm diameter at tip
TP1	6.3 nm ± 0.5 nm	5.83 nm ± 0.6 nm	24.8 nm ± 2.3 nm	5.3 nm
TP2	10.9 nm ± 0.8 nm	N/A (~same as base)	41.3 nm ± 4.6 nm	N/A (~same as base)
TP3	8.2 nm ± 1.3 nm	7.12 nm ± 1.4 nm	40.7 nm ± 3.8 nm	6.4 nm
TP4	10.6 nm ± 2.4 nm	9.46 nm ± 2.0 nm	41.3 nm ± 4.6 nm	8.8 nm

Supplementary Note 2: Single-tetrapod optical measurements

To prepare samples for single tetrapod investigation, a highly dilute solution of tetrapods in hexanes was dropcast onto a clean glass coverslip to create a density of ~0.02 tetrapod per μm^2. Blinking and photobleaching measurements were carried out in a home built wide-field optical microscope. Briefly, a 405 nm, 50 mW CW laser was focused via an optical lens on the sample surface with a spot of ~150 μm in diameter, affording a power density of ~10 W/mm^2. The fluorescence emission was collected with a LMPLFLN 50X/NA0.50 objective lens (Olympus). Typically, ~20-30 QDs were observed in the field of view. With a LN-cooled CCD camera, a series of wide-field fluorescence images were recorded with an integration time of 0.1 s per frame over 1 h.

Measuring $g^{(2)}$ values and lifetimes of single tetrapods. A 405 nm pulsed laser (~80 ps pulse width) and a laser scanning confocal microscope equipped with a 100×, 0.85 NA objective were used to excite the tetrapods. Photoluminescence from single tetrapods was collected by the same objective lens and then directed to two avalanche photo diodes (APDs) forming a Hanbury-Brown-Twiss spectrometer. This allowed for identification of single nanostructures *via* determination of single-photon emission behavior of the low-energy emission under low excitation power density

($\{N\} << 1$). For measurements of single tetrapod lifetime, $g^{(2)}$, and most spectra, the repetition rate was 1 MHz. For the high-power single tetrapod spectra measurement, the repetition rate was chosen to be 3 MHz to enhance excitation of multiexcitons. To identify dual-emitting tetrapods, switchable 510 nm short-pass filters and 610 nm long-pass filters were installed in front of the two APDs. With different filters before the two APDs, a quick dual-channel scan identifies emissive spots that radiate strongly at both short band and long band simultaneously.

To obtain increasing pump fluences of 13, 38 and 116 μJ/cm^2 per pulse for power-dependence measurements, the same single tetrapod was investigated at each power, beginning with the lowest power. The laser power was then adjusted to increase the measured power at the sample for each subsequent measurement. Low-frequency excitation (Figure 4, main text) was performed at 1 MHz, while high frequency excitation (Figure 6d, main text) was performed at 3 MHz. Spectra were recorded by dispersing the collected single tetrapod emission onto a CCD array using a Princeton Instruments Acton SP2300 Spectrometer. Single-tetrapod data were collected by averaging three spectra that were integrated for 30 s each.

To further probe Auger recombination efficiency in the TP4 tetrapods, we compare $g^{(2)}$ and time-gated $g^{(2)}$ values for red core emission (main text Figure 6c) as a means for temporally separating emissions from excitonic and biexcitonic states.[8,9] Specifically, we find that the normalized area of the center peak of the $g^{(2)}$ spectrum [$g^{(2)}(0)$] before time-gating is >0, i.e., 0.39 (Figure 6c, left), while after time-gating, $g^{(2)}(0)$ diminishes to ~0 (Figure 6c, right). This observation not only allows us to confirm that we are interrogating a single tetrapod (i.e., if multiple tetrapods were contributing to the PL signal, then the time-gated $g^{(2)}$ value would not have diminished), but also to assess the efficiency of biexciton emission and, thereby, the extent of Auger recombination suppression.[8,9] The un-gated $g^{(2)}(0)$ is a measure of the relative biexciton

to exciton emission efficiency, i.e. the value 0.39 indicates that for this tetrapod biexciton emission efficiency is approximately half that of the exciton emission efficiency. Together with the observation of clear signatures of room-temperature multiexcitonic emission (see main text), this high biexciton efficiency further confirms that Auger recombination is suppressed. Finally, Auger suppression may also be responsible for reduced fluorescence intermittency in these tetrapods.[10] The moderate blinking suppression observed in on-time histograms (main text Figure 2d) and stability in the single-tetrapod PL-intensity/time trajectory (corresponding to the described $g^{(2)}$ behavior) (main text Figure 6a) are also consistent with the conclusion that Auger recombination has been suppressed in the CdSe core.

Extended discussion on the nature of dual-red PL in CdSe/CdS tetrapods. These references differ with respect to which red PL peak is assigned to the direct or indirect carrier recombination process, respectively. Shafran utilized tetrapods similar to our TP1 (thin/short) sample (4 nm CdSe core with CdS arms 6 nm in diameter and 25 nm in length) and provided both excitation and polarization-dependent data to conclude that *indirect* carrier recombination was responsible for the redder of the two red PL peaks. We come to a similar conclusion based in part on our observation that the predominant red PL peak in our ensemble tetrapod measurements and in most single-TP spectra is significantly red-shifted from the starting CdSe core emission (~650 nm compared to 605 nm), a characteristic of reduced carrier confinement afforded by a quasi type II band alignment (**Supplementary Figure 4b**).

The indirect state as pictured in **Supplementary Figure 4b** is the lowest energy state, as the CdS conduction band level as shown is lower in energy than the CdSe conduction band level. On the other hand, quasi type-II band alignment can also be considered as resulting from near alignment of either the conduction or valence bands (in the case of CdSe/CdS, the conduction

bands are nearly aligned). In the alternative view, CdSe/CdS indirect recombination involves a partially delocalized electronic state with a fully core-localized hole state. In either case, for CdSe core emission to compete with indirect emission, an interfacial potential-energy barrier should be present (**Supplementary Figure 4b**).[11] This barrier can slow the distribution of core conduction-band electrons to shell-dominated states, affording an opportunity for direct core-localized electron-hole recombination.

Single tetrapod intensity saturation curves. The intensity saturation curves shown in Supplementary Figure 1 were created by measuring the intensity response to varying power for a single tetrapod under pulsed 405 nm excitation at 1 MHz. Once a single tetrapod was in focus, the intensity was swept from low to high and back in an oscillating linear pattern with a period of 30 s for a total measurement time of 5 min. Vertical distribution of data points is due to data being collected from the same single TP at the same pump power, but at different times during the measurement. Variations in intensity arise due to blinking, flickering, and statistical fluctuations of the single TP intensity. This provides a comprehensive view of the effect of PL intensity fluctuation on the saturation of each single TP.

Fluorescence lifetime-intensity distribution (FLID) diagrams and blinking traces for single tetrapods. To calculate the number of excitons generated per photon for the blinking traces shown in Figure 3, the PL intensity was first divided by the absorption cross-section of each separate TP sample. Then, the trace intensity was corrected for differences in beam power. Finally, all traces were normalized to the highest intensity point shown in the trace for TP1 (a binary blinking TP), which is expected to have a "bright" state with PLQY ≈ 1. This allows comparison of blinking traces intensity across TP samples. FLID diagrams were generated with a bin time of 100 ms. The FLIDs shown in Figure 3 represent single tetrapod measurements of ~5 min.

Calculating absorption cross-section and number of excitons generated. To create the top axis of the tetrapod intensity saturation curves, the number of excitons formed in each tetrapod was estimated based on excitation fluence and tetrapod absorption cross-section. We used the relation N = jσ, where N is the number of excitons generated, j is the per-pulse laser intensity in photons/cm^2 per pulse, and σ is the absorption cross-section of the tetrapod in cm^2.

The tetrapod and core-only absorption cross-sections were calculated, with volume as the basis, using known methods previously used for CdSe/CdS QDs.[12] The absorption cross-section, σ_w, is calculated using the equation,

$$\sigma_w = V\alpha_w|f_w|^2 \frac{n_w}{n_{medium}} \qquad \text{Equation 1}$$

where V is the volume of the tetrapod, α_w is the absorption coefficient of CdS, f_w is a correction factor for local field effects, n_w is the refractive index of CdS, and n_{medium} is the refractive index of air. We approximate the heterostructure composition as 100% CdS because, as noted by Park *et al*, the core contribution to the total cross-section is small and the optical constants of CdSe and CdS are similar; therefore, whether the core is considered to be CdSe or CdS, the resulting value for α_w does not change.

Supplementary Note 3: Ensemble photoluminescence-decay measurements

Thick films of each tetrapod sample were created by drop-casting solutions of moderate optical density (O.D. ~0.2) onto a clean glass coverslip. Ensemble PL decay was recorded for each sample using the optical microscope described above. A repetition rate of 250 kHz was used to ensure no multiexciton generation, and the excitation power was decreased so that the total counts on each detector were < 1% of the repetition rate (2500 cps). Single photon events were collected for ~10-20 min and histogrammed to yield the ensemble PL decay curves shown in the main text.

PL saturation of single tetrapods. Supplementary Figure 1 shows the PL saturation behavior of a single tetrapod from each of the examined samples. TP1 shows a linear response to pump fluence in the regime studied here; this is because the volume (and therefore the absorption cross-section) of TP1 is small in comparison to other tetrapods, so the number of excited e-h pairs is also small. Still, it is evident in all tetrapods that saturation of PL occurs well past generation of 1 exciton in the tetrapod. This is because, as discussed in the main text, most electron-hole pairs are formed in the tetrapod arms, and few of the electron-hole pairs generated in the CdS arm relax to the CdSe core for radiative emission. All green emission shown from single tetrapods occurs well past the point of saturation, confirming that the green arm emission and core PL broadening seen in Figure 4 are multiexcitonic processes.

Lifetime level analysis to confirm A-type blinking in TPs. In main text Figure 3, it is clear from the shape of the FLID that TP2 and TP4 display A-type blinking: a reduction in PL intensity occurs simultaneously with shortening of the PL decay lifetime. This effect is more subtle in the FLIDs for TP1 and TP3. For this reason, we provide further analysis of the relationship between intensity and decay lifetime for these two TPs. In Supplementary Figure 5, all photons within the red(blue) shaded region are analyzed to determine the lifetime of the high(low) intensity emissive states of the TPs. From the decay curves for these regions (shown in Supplementary Figure 5b and Supplementary Figure 5d) it can be seen that the lifetime varies with intensity in these TPs, confirming that the emission from these TPs is influenced by Auger-mediated charged emission.

Supplementary Note 4: Transient absorption spectroscopy

The accuracy of the approach is limited by the time resolution of the TA experiment. In the present case the measured instrument response function was FWHM = 220 fs. As a result, the Auger

relaxation of some of the higher order multi-excitons (N_{e-h}>5), which are likely to decay with time-constant t<220 fs is likely not resolved in the transient signal. This may lead to underestimation of the total number of excitons in the analysis. However, for the N_{e-h}<5, where time constant is likely to be >220 fs the error is expected to be small.

Supplementary Table 3. Summary of the results of the numerical fitting of the experimental single-tetrapod PL data for samples TP1, TP2, TP3 and TP4 and of the spectral shifts obtained from fitting results. (I(rel) = Integrated PL intensity of a band as a fraction of total integrated observed PL.)

In nanometers

	13 uW											
	Peak 1			Peak 2			Peak 3			Peak 4		
	λ (nm)	I (rel)	σ (nm)	λ (nm)	I (rel)	σ (nm)	λ (nm)	I (rel)	σ (nm)	λ (nm)	I (rel)	σ (nm)
**TP1**	646.6	1.00		-	-		-	-	-	-	-	-
**TP2**	648.0	1.00		-	-		-	-	-	-	-	-
**TP3**	655.5	0.96	35.70	618.8	0.04	25.20	-	-	-	-	-	-
**TP4**	664.4	0.75	39.50	646.8	0.16	25.20	633.1	0.07	33.50	497.6	0.03	39.20

	38 uW											
	Peak 1			Peak 2			Peak 3			Peak 4		
	λ (nm)	I (rel)	σ (nm)	λ (nm)	I (rel)	σ (nm)	λ (nm)	I (rel)	σ (nm)	λ (nm)	I (rel)	σ (nm)
**TP1**	648.1	1.00		-	-		-	-	-	-	-	-
**TP2**	649.0	0.90	29.20	621.6	0.04	25.80	-	-	-	494.6	0.06	37.1
**TP3**	656.0	0.79	33.70	622.7	0.08	34.60	592.4	0.04	39.9	498.0	0.08	40.0
**TP4**	667.9	0.54	40.00	645.6	0.30	31.80	618.0	0.08	39.60	497.3	0.08	26.8

	116 uW											
	Peak 1			Peak 2			Peak 3			Peak 4		
	λ (nm)	I (rel)	σ (nm)	λ (nm)	I (rel)	σ (nm)	λ (nm)	I (rel)	σ (nm)	λ (nm)	I (rel)	σ (nm)
**TP1**	650.7	1.00		-	-		-	-	-	-	-	
**TP2**	648.6	0.77	28.20	615.2	0.09	31.70	-	-	-	493.1	0.15	40.0
**TP3**	655.8	0.61	39.60	629.3	0.14	40.00	592.4	0.10	39.90	496.5	0.15	38.2
**TP4**	671.0	0.34	40.00	642.2	0.35	39.90	606.1	0.12	39.70	497.2	0.18	20.4

	13uW		38uW		116uW	
**Shifts**	ΔP1P2 (meV)	ΔP1P3 (meV)	ΔP1P2 (meV)	ΔP1P3 (meV)	ΔP1P2 (meV)	ΔP1P3 (meV)
**TP1**	-		-		-	
**TP2**	-		84.2		103.7	
**TP3**	112.2		101.1	202.9	79.6	202.3
**TP4**	50.8	92.3	64.1	149.9	82.9	197.9

Supplementary Table 4. Calculated number of photons absorbed by the arms and the core (average <N_{abs}>) as a function of pump fluence for tetrapod TP2 ('short' and 'thick').

	Optical Constant (cm2/nm3)	Volume (nm3)	Abs. CS (cm2)	Incident Np (@0.013mJ)	<Nabs> (0.013mJ)	Incident Np (@0.038mJ)	<Nabs> (0.038mJ)	Incident Np (@0.116mJ)	<Nabs> (0.116mJ)	Incident Np (@0.25mJ)	<Nabs> (0.25mJ)
**CdS (arms)**	8.36E-17	10301.8	8.61E-13	2.56E+13	22.05	7.69E+13	66.15	2.31E+14	198.46	5.12E+14	441.0
**CdSe (core)**	8.19E-17	33.5	2.74E-15	2.56E+13	0.07	7.69E+13	0.21	2.31E+14	0.63	5.12E+14	1.4

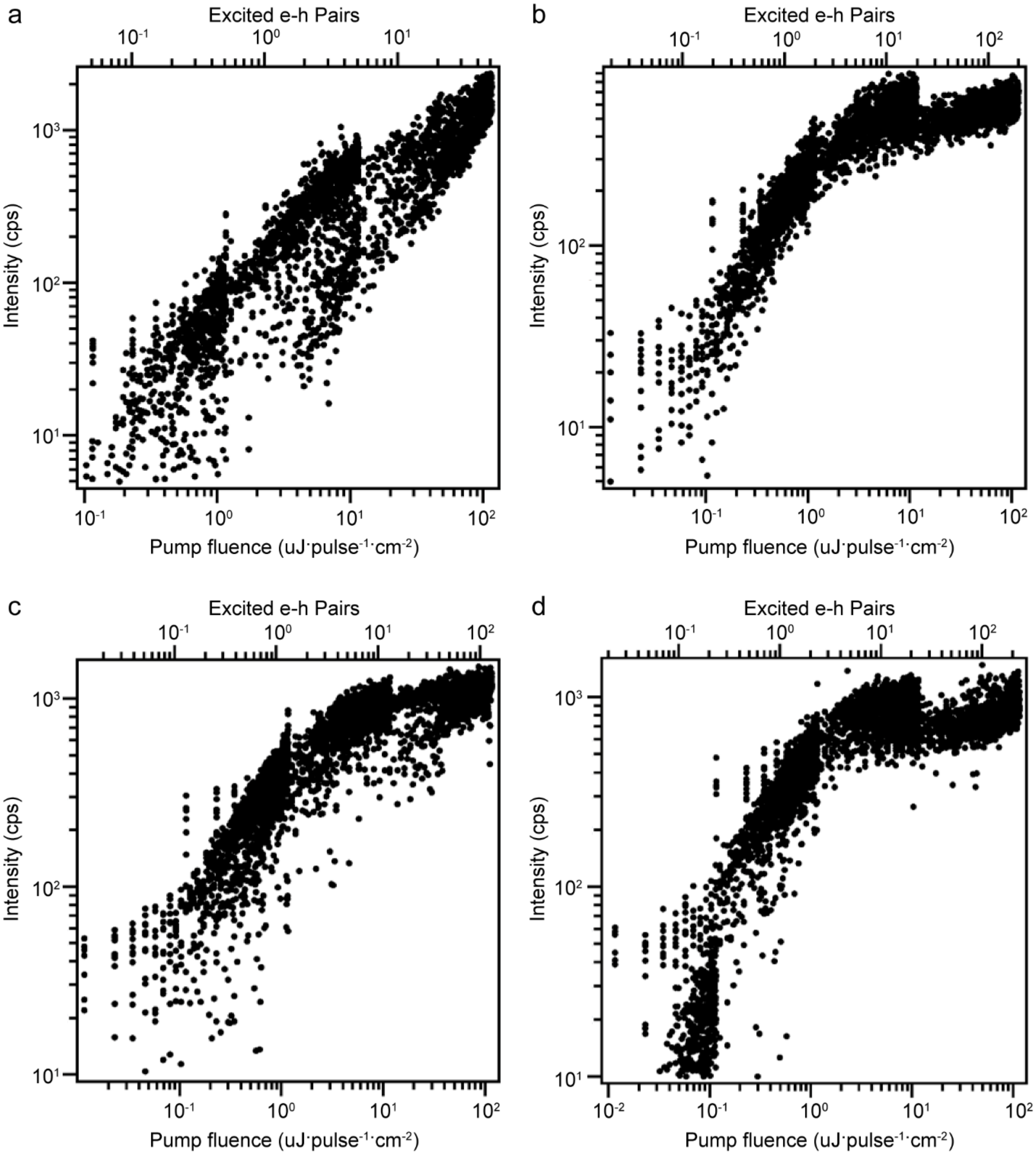

Supplementary Figure 1. PL saturation curves. Intensity *vs* pump fluence for single tetrapods from sample TP1 (a), TP2 (b), TP3 (c), and TP4 (d). The top axis represents the expected (calculated) number of electron-hole pairs present in the entire tetrapod volume based on the absorption cross-section of the tetrapod.

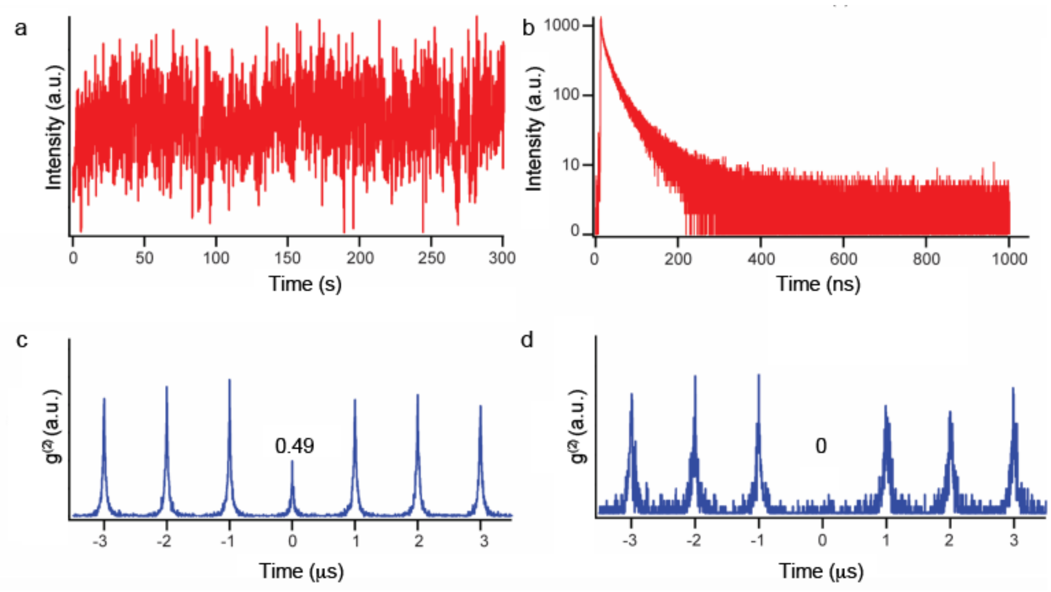

Supplementary Figure 2. Single-tetrapod optical properties for thick/short-arm tetrapods (TP2). (a) Single-tetrapod intensity-time trace reveals non-blinking and non-photobleaching behavior characteristic of thick/thick-arm tetrapods (TP2; main text). (b) Photoluminescence decay curve for the CdSe core emission. (c) Second-order autocorrelation function ($g^{(2)}$) spectrum. (d) Time-gated $g^{(2)}$, for which the normalized area of the center peak is zero, indicating that the non-zero area shown in (c) results from contributions from bi/multiexciton emission (see discussion in main text).

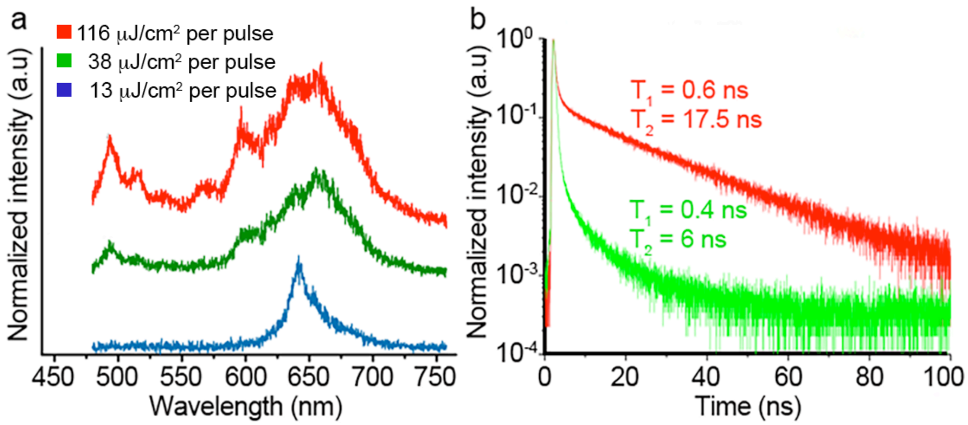

Supplementary Figure 3. Two-color emission in thick and long-arm tetrapods (TP4). (a) Pump fluence-dependent spectra for a single TP4 (thick/long-arm) tetrapod reveal emergence of both multiexcitonic dual red/green emission and emission from multiexcitonic and charged states in the core and arms with increasing pump fluence. The repetition rate here was chosen to be 3 MHz to enhance excitation of multiexcitons, compared to 1 MHz. (b) Spectrally separated PL decay curves for the CdSe core emission (red) and the CdS arm emission (green) at fluence of 116 $\mu J/cm^2$ per pulse excitation.

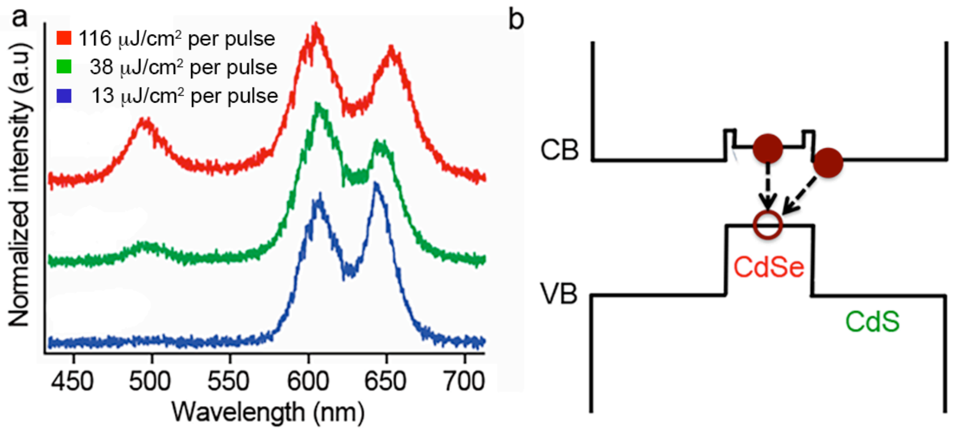

Supplementary Figure 4. Tri-color emission in thin and long-arm tetrapods (TP3). (a) Pump fluence-dependent spectra for a single TP3 (thin/long arm) tetrapod reveal dual red emission at all pump fluences and emergent green PL at higher pump fluences. (b) Diagram depicting CdSe-core/CdS-arm valence and conduction band (VB and CB, respectively) alignments characteristic of a quasi type-II electronic structure (here: strong localization of hole to CdSe core and weak localization of electron to CdS shell or delocalization of electron between core and shell), to which an interfacial energy barrier has been added to show how an excited-state electron might be confined to the core to induce direct excitonic emission in addition to indirect (quasi type-II) emission (i.e., dual red PL) .

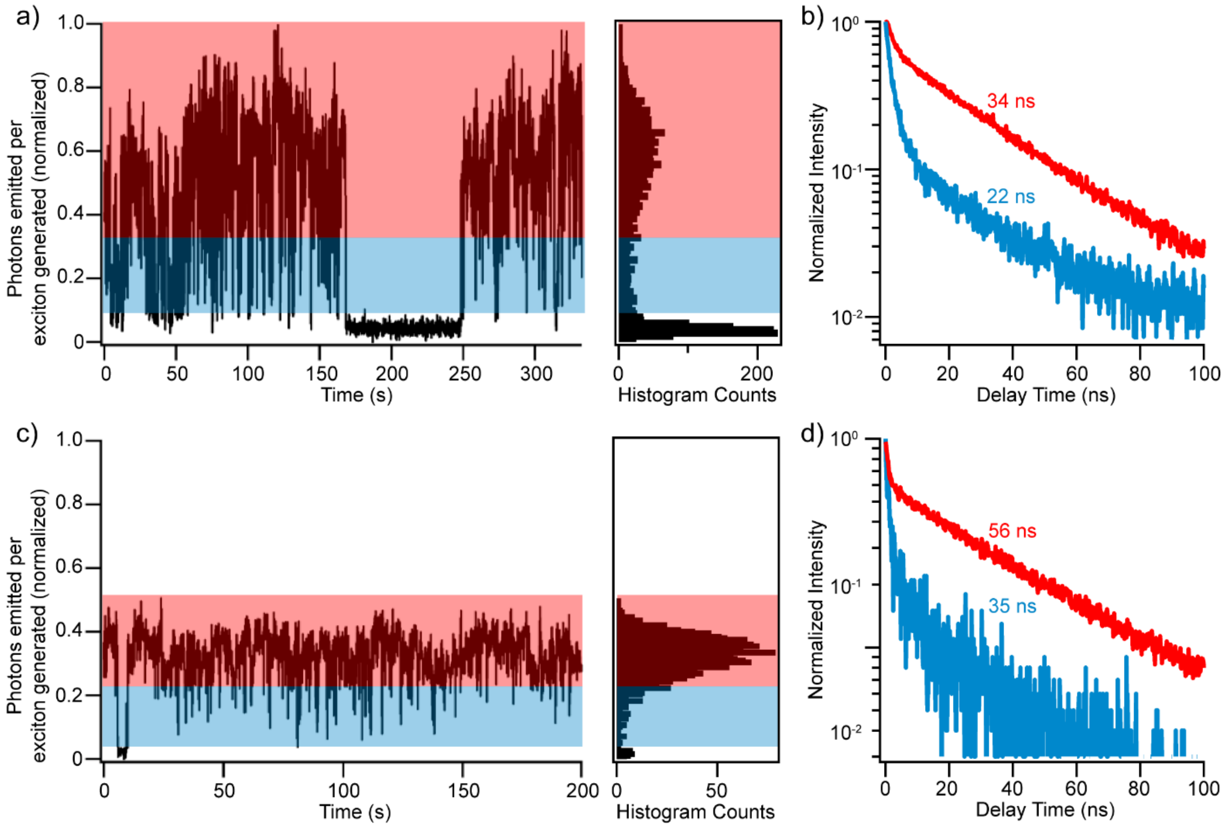

Supplementary Figure 5. Further analysis of A-type blinking in TP1 and TP3. Shown in (a) is the blinking trace for TP1 with "bright" and "dim" states highlighted in red and blue, respectively. Extracting the PL decay lifetime for only photons in the "bright" and "dim" states demonstrates that lower intensity states correspond with shorter lifetimes (b). The blinking trace (c) and lifetimes (d) for TP3 show the same intensity-dependent lifetime behavior.

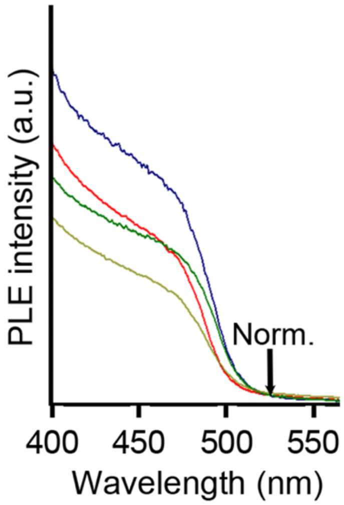

Supplementary Figure 6. PL excitation (PLE) spectra for each tetrapod of the size-series [TP1 (red), TP2 (ochre), TP3 (green) and TP4 (blue)]. PLE spectra were obtained for red emission in hexane and normalized to the same intensity at 525 nm. Tetrapod suspensions were somewhat turbid (with the exception of TP2), causing scattering in absorption measurements and making it not possible to report "equivalent-concentration" solutions based on absorption optical density. For this reason, we compare PLE spectra as these are less affected by scattering. Spectra were normalized at a wavelength (energy) below the bandgap of the CdS arms (bulk bandgap: ~515 nm or 2.42 eV), i.e., where only the CdSe cores can absorb light and contribute to PL. In this way, we account for effects of different TP concentrations. In other words, we account for solution concentration by normalizing to the CdSe core PLE intensity, as identical cores were used to synthesize each of the four tetrapod geometries. Thus, for a given CdSe core PLE, the PLE intensity can be compared from 400-515 nm, which includes contributions from the CdS arms, i.e., both CdSe core and CdS arms can absorb excitation photons in this spectral region and potentially contribute to PL. The observation that PLE intensity trends do not necessarily follow absorption cross-section values (i.e., tetrapod volume trends) suggests that the relative effectiveness of particular arm geometries in harvesting excitation energy for red core emission

depends on more than size, cross-section or volume. As described in the main text, we were able to quantitatively assess the arm-to-core transfer for TP2 (non-scattering tetrapod suspension) using transient absorption spectroscopy.

Supplementary References

(1) Yang, Y. A.; Wu, H.; Williams, K. R.; Cao, Y. C. *Angew. Chem. Int. Ed Engl.* **2005**, *44* (41), 6712-6715.

(2) Leatherdale, C. A.; Woo, W.-K.; Mikulec, F. V.; Bawendi, M. G. *J. Phys. Chem. B* **2002**, *106* (31), 7619-7622.

(3) Mishra, N.; Lian, J.; Chakrabortty, S.; Lin, M.; Chan, Y. *Chem. Mater.* **2012**, *24* (11), 2040-2046.

(4) Mishra, N.; Wu, W.-Y.; Srinivasan, B. M.; Hariharaputran, R.; Zhang, Y.-W.; Chan, Y. *Chem. Mater.* **2016**.

(5) Talapin, D. V.; Nelson, J. H.; Shevchenko, E. V.; Aloni, S.; Sadtler, B.; Alivisatos, A. P. *Nano Lett.* **2007**, *7*, 2951-2959.

(6) Li, J. J.; Wang, Y. A.; Guo, W.; Keay, J. C.; Mishima, T. D.; Johnson, M. B.; Peng, X. *J. Am. Chem. Soc.* **2003**, *125*, 12567-12575.

(7) Ghosh, Y.; Mangum, B. D.; Casson, J. L.; Williams, D. J.; Htoon, H.; Hollingsworth, J. A. *J. Am. Chem. Soc.* **2012**, *134*, 9634-9643.

(8) Nair, G., Zhao, J. & Bawendi, M. G. Biexciton Quantum Yield of Single Semiconductor Nanocrystals from Photon Statistics. *Nano Lett.* **2011,** *11*, 1136-1140.

(9) Park, Y.-S. *et al.* Near-Unity Biexciton Emission Quantum Yields in Individual CdSe/CdS Nanocrystals Revealed by Two-Photon-Correlation and Photoluminescence-Saturation Measurements. *Phys. Rev. Lett.* **2011**, *106,* 187401.

(10) Galland, C. *et al.* Two types of luminescence blinking revealed by spectroelectrochemistry of single quantum dots. *Nature* **2011**, *479*, 203-207.

(11) Shafran, E., Borys, N. J., Huang, J., Talapin, D. V. & Lupton, J. M. Indirect Exciton Formation due to Inhibited Carrier Thermalization in Single CdSe/CdS Nanocrystals. *J. Phys. Chem. Lett.* **2013**, *4*, 691-697.

(12) Park, Y.-S. et al. Near-Unity Biexciton Emission Quantum Yields in Individual CdSe/CdS Nanocrystals Revealed by Two-Photon-Correlation and Photoluminescence-Saturation Measurements. *Phys. Rev. Lett.* **2011**, *106*, 187401.
